# *Raphia*-Microorganism Composite Biosorbent for Lead Ion Removal from Aqueous Solutions

**DOI:** 10.3390/ma14237482

**Published:** 2021-12-06

**Authors:** Paweł Staroń, Jarosław Chwastowski

**Affiliations:** Department of Engineering and Chemical Technology, Cracow University of Technology, 24 Warszawska Str., 31-155 Cracow, Poland; jaroslaw.chwastowski@pk.edu.pl

**Keywords:** *Saccharomyces cerevisiae*, immobilization, adsorption, biosorption, equilibrium, kinetic, yeast

## Abstract

This study investigated the possibility of obtaining a *raphia*-microorganism composite for removing lead ions from aqueous solutions using immobilized yeast cells *Saccharomyces cerevisiae* on *Raphia farinifera* fibers. The obtained biocomposite was characterized using scanning electron microscopy and Fourier transform infrared spectroscopy. Studies were conducted to determine the influence of contact time, initial concentration of Pb(II), and pH allowed for the selection of nonlinear equilibrium and kinetic models. The results showed that the biocomposite had a better Pb(II) removal capacity in comparison to the *raphia* fibers alone, and its maximum Pb(II) adsorption capacity was 94.8 mg/g. The model that best describes Pb(II) sorption was the Temkin isotherm model, while kinetic studies confirmed the chemical nature of the sorption process following the Elovich model. The obtained research results provide new information on the full use of the adsorption function of biomass and the ubiquitous microbial resources and their use in the remediation of aqueous environments contaminated with heavy metals.

## 1. Introduction

In this era of strong industrial development and increased human activity, water pollution has become a major global problem. In addition, water is an essential element of life on earth, and its storage is decreasing year by year [1,2]. One of the main water pollutants is heavy metals, which have a negative impact on the environment. This is due to their toxic properties in relation to living organisms, and additionally, they are not biodegradable, which causes several environmental problems [3].

Primary pollutants come from burning fossil fuels, fertilizers, pesticides, municipal waste, mining and smelting of ferrous ores, industrial waste, oil spills, nuclear waste spills [4,5]. Heavy metals such as lead, nickel, and cadmium have become ecotoxicological hazards of primary importance due to their tendency to accumulate in vital organs of humans and animals (e.g., liver, kidney, brain).

Commonly used processes for removing heavy metal ions from wastewater include chemical precipitation and filtration, chemical oxidation or reduction, electrochemical treatment, reverse osmosis, ion exchange and evaporation [6]. However, these methods are not ideal due to the need for complex instruments and space-consuming equipment, and they generate high maintenance costs, which affect the economics of the process [7]. From an economic perspective, a more promising approach is to use adsorption or photocatalysis processes to remove heavy metals from water and wastewater [8,9]. Currently, widely used adsorbents in the removal of pollutants from water and wastewater include activated carbon, zeolites and various types of clays and aluminophosphates and photocatalysts such as titanium dioxide and metal sulfides. The adsorbents and photocatalysts used are unfortunately not without disadvantages. Among the main disadvantages of the adsorbents mentioned are small surface area and lack of selectivity, while in the case of photocatalysts, it is the low efficiency of sunlight and poor quantum yield of photocurrent [10]. Therefore, there is still a great need to develop new and better adsorbents and/or photocatalysts for water and wastewater treatment. Research in recent years has indicated that biosorption is one of the most promising technologies for removing heavy metal ions from water and wastewater. Various types of bio-based materials are widely used, including wastes from households or other industries, including agriculture. Food crops are grown worldwide (e.g., sugarcane, rice, corn, wheat, etc.), and parts other than fruits, grains, juices, etc., are available for biosorption experiments [11]. In addition to the use of dead biomass as heavy metal sorbents (e.g., coconut fiber, peat, wool, sawdust, straw), there are many literature reports on the use of microorganisms (live biomass) that can remove and/or accumulate large amounts of heavy metals [12]. Among these microorganisms, bacteria, fungi, and algae are the most widely used. Several bacterial-based heavy metal remediation methods have been investigated, including *Escherichia*, *Pseudomonas*, *Bacillus* and *Micrococcus*, fungi, e.g., *Aspergillus niger*, *Aspergillus fumigatus*, *Termitomyces clypeatus*, *Saccharomyces cerevisiae*, algae, e.g., *Fucus vesiculosus*, *Cladophora fascicularis*, *Cladophora fascicularis*, *Cystoseira crinitophylla*, *Saccharina japonica*, and *Sargassum fusiforme* [13].

The use of microorganisms to remove heavy metals from water and wastewater has advantages but also disadvantages. The main disadvantages include mechanical instability and the difficult separation of biomass after the removal process. To eliminate these disadvantages, various methods are used, including the method of immobilization of microorganisms on the carrier. This allows for increased productivity, improved mechanical strength, and increased chemical resistance. Different types of immobilization have been defined, including by encapsulation or trapping, among others [14]. Encapsulation is associated with the use of semi-permeable membranes, while trapping involves the immobilization of cells in a gel matrix, typically having a bead shape. There are several matrices used to immobilize a given biosorbent containing microbial cells, which include calcium alginate, silica, or polymers such as polyurethanes and polyacrylamide. A particularly important aspect is the use of a properly selected matrix, as it determines the chemical resistance and mechanical strength of the biosorbent. The matrix should be, above all, inexpensive and feasible to produce [15]. The higher biodegradation efficiency observed after the use of immobilized microorganisms in comparison to free ones has led to an increased interest in their application in bioremediation processes [16]. To improve the properties of the obtained composites, a carrier may be used on which microorganisms will be immobilized. The carriers are classified as organic and inorganic or natural or synthetic. Examples of the use of biocomposites to remove contaminants from water and wastewater are: yeast cells immobilized on coconut fiber for lead removal [17], biocomposite materials for Cr (VI) removal in water by immobilization of a previously isolated *Morganella morganii* strain [18], removal of chromium ions by agar immobilized cells of the cyanobacterium *Anacystis nidulans* [19], nanocellulose-based biocomposite coupled with *Arthrobacter globiformis* as a herbicide degrader for the rapid elimination of diuron [20], bio-composite of Fe-sludge biochar and *Bacillus sp* for decolorization of Methylene blue dye [21].

The aim of this study was to obtain a composite material constructed from a carrier of natural origin and yeast as a biosorbent for lead ion removal. *Raphia (Raphia farinifera*) fibers, whose main components are cellulose, hemicellulose, and lignin, was used as the carrier. *Raphia* fibers have been successfully used as a biosorbent to remove ammonia and methylene blue, confirming its ability to remove pollutants from the aquatic environment [22,23]. The biomass used was the yeast *Saccharomyces* *cerevisiae*, which is one of the most common microorganisms available in large quantities as a by-product of fermentation processes or the pharmaceutical industry. *Saccharomyces cerevisiae* has been used by several researchers as a biosorbent to remove or accumulate heavy metals such as copper, cadmium, lead and mercury from wastewater [6]. Sodium alginate immobilization was used as the immobilization method for microorganisms and calcium chloride, which has high biocompatibility, non-toxicity, and simple gelation properties, was used as the crosslinking agent (Zhao et al. 2019). Lead ion removal studies were conducted in batch-type periodic systems. The results obtained determined the maximum sorption capacity of the *Raphia-Saccharomyces cerevisiae* (RA-SC) composite for lead ions and compared them with *raphia* fibers. Using nonlinear equilibrium and kinetic models, the mechanism of the sorption process was determined.

## 2. Materials and Methods

### 2.1. Materials and Chemicals

The yeast *Saccharomyces cerevisiae* (SC) (purchased from the National Collection of Yeast Cultures, Norwich, UK), sieved under sterile conditions into pre-sterilized 250 cm^3^ conical flasks containing YEPD (Yeast Extract Peptone Dextrose Agar) medium, was used to prepare the composite sorbent. The culture was conducted using a laboratory shaker at 25 °C for 72 h. After this time, the medium in the flask became milky and a precipitate formed at the bottom indicating significant growth of *Saccharomyces cerevisiae* in the flask. In this study, an inoculum containing *S. cerevisiae* in a population of approximately 1.5 · 10^10^ CFU/cm^3^ was used. The yeast inoculum (80 cm^3^) was centrifuged for 15 min at 10,000 RPM using an MPW-260R centrifuge (MPW Med. Instruments, Warsaw, Poland).

Natural *raphia* (RA) purchased from a commercial store had a fiber width of 5 mm and a total length of 1–1.2 m. Prior to testing, the material was cleaned of impurities by washing it in deionized water for 30 min and then rinsing it 3 times with deionized water. The cleaned and 70 °C dried *raphia* was ground using a RETSCH^®^ MM400 Mixing Mill (Retsch GmbH, Haan, Germany) (vibration frequency: 30 Hz, grinding time: 5 min) and then passed through a sieve (Mesh 42). The material thus prepared was designated as RA and used in sorption studies.

Reagents used in the research: YEPD Agar, sodium alginate, calcium chloride, potassium dichromate (VI), lead (II) chloride, cobalt (II) chloride hexahydrate, zinc (II) nitrate hexahydrate, nitric acid (V), sodium hydroxide were characterized by high purity and were from Sigma-Aldrich. All solutions were prepared with deionized water.

### 2.2. Preparation of RA-SC

To obtain the RA-CS composite, 2 g of sodium alginate were added to 98 cm^3^ of deionized water and heated in a water bath to 90 °C. Then, the mixture was stirred for 2 h to completely dissolve it. After a homogeneous mixture was obtained (no lumps), 1.0 g of *raphia* was added and stirring was continued until a homogeneous mixture was obtained. The *raphia* concentration in sodium alginate was 10 g/dm^3^. The use of *raphia* improved the mechanical properties and stability of the resulting composite, and as a material of biological origin, it complies with the principles of green chemistry and sustainable development. Then yeast (obtained by centrifugation of 80 cm^3^ inoculum) was added. The whole content was stirred at a temperature of 30 °C for 1 h. The mixture prepared in this way was added dropwise with the use of an injector to a solution of calcium (II) chloride with a concentration of 2%. The resulting beads were left in the solution for 10 min. After this time, the resulting RA-SC composite was washed several times with deionized water, and then dried by lyophilization. The dried RA-SC composite was stored at 4 °C for further experiments.

### 2.3. Characterization

The surface morphology of RA and RA-SC before and after the lead ion removal process was observed using a scanning electron microscope Hitachi TM-3000 (Hitachi High-Technologies Corporation, Tokyo, Japan) equipped with an X-ray microanalyzer EDX Bruker Quantax 70 (Bruker, Berlin, Germany), and EDX spectroscopy was used to detect the elements present, including Pb present before and after sorption. FTIR spectroscopy was performed to confirm the presence of functional groups on the SC, RA and RA-SC surfaces before and after the sorption process in the wavenumber range 400–4000 cm^−1^ Thermo Scientific—Nicolet iS5 with the ATR iD7 attachment (Thermo Fisher Scientific, Dublin, Ireland). A specific surface area test was carried out using an ASAP 2010 deaerator station. Before measurement, samples were dried in a helium atmosphere at 110 °C for 8 h, then under vacuum at 100 °C and 0.001 Tor for 8 h.

### 2.4. Sorption Process

#### 2.4.1. Choosing the Appropriate Metal Ions

All heavy metals sorption processes were carried out in batch-type periodic systems. Sorption experiments were carried out in 60 cm^3^ closed PP containers. The assumption of the selection of heavy metal for the research was to determine the element with the highest affinity for raw RA and RA-SC, which allowed to compare the removal ability of this element by RA and the RA-SC composite obtained based on RA. Pb(II), Co(II), Cr(VI), Zn(II) ions were selected for the research and model solutions of these ions were prepared at a concentration of 500 mg/dm^3^.

The sorption process was carried out, 0.1 g of RA/RA-SC was weighed into the containers, then 20 cm^3^ of the model solution of individual ions were added and mixed at a constant speed at 25 °C for 3 h. After the process was completed, the mixture was separated by filtration. The filtrate was analyzed on an acetylene air-flame atomic absorption spectrometer (F-AAS). The amount of adsorbed metals on the tested adsorbent was calculated from Equation (1) (Table 1).

On the basis of the obtained results, it was found that the lead ions showed the highest affinity for RA and RA-SC. The sorption capacity for other metals is much lower and is in the following series: Co^2+^ < Zn^2+^ < Cr^6+^ < Pb^2+^ (Pb^2+^: RA—16.51 mg/g, RA-SC—94.66 mg/g; Cr^6+^: RA—6.16 mg/g, RA-SC—63.25 mg/g, Zn^2+^: RA—0.60 mg/g, RA-SC—12.83 mg/g, Co^2+^: RA—0.00 mg/g, RA-SC—5.42 mg/g). Subsequently, the research focused on lead ions.

#### 2.4.2. Specific Sorption Studies

In sorption studies, the influence of variables including time, initial concentration of Pb(II) and pH value was checked. 0.1 M HNO_3_ and 0.1 M NaOH were used to achieve the intended pH values. The pH values were equal to 3, 4, 5 and 6 respectively. pH values below 3 were not used due to the excessive protein denaturing properties in the microbial cells [27]. The research on the effect of pH on the value of sorption capacity showed that with the increase of pH from 3.0 to 5.0, the sorption capacity increased, while above 5 it began to decline (pH = 3: RA—5.21 mg/g, RA-SC—61.08 mg/g; pH = 4: RA—12.32 mg/g, RA-SC—76.42 mg/g, pH = 5: RA—16.51 mg/g, RA-SC—94.66 mg/g, pH = 6: RA—13.91 mg/g, RA-SC—73.12 mg/g). The obtained results were consistent with the research conducted by [28], which confirms that H^+^ ions compete with metal ions at low pH, and the metal ion binding sites are only available in the deprotonated state. Therefore, as the pH increases, the density of the positive charge on the surface of the material decreases, so that the protonation effect becomes smaller, which results in greater availability of metal-binding sites [29]. The lead ion sorption process was carried out at the pH corresponding to the highest Pb(II) sorption, i.e., pH = 5 at 25 °C using a laboratory shaker with 150 rpm for various concentrations of Pb(II)—100, 200, 300, 400 and 500 mg/dm^3^ for 0.5, 1, 3, 5, 8, 15 and 30 min. 0.1 g of RA or RA-SC was weighed into PP containers, to which 20 cm^3^ of the solution with a given starting concentration of Pb(II) was added each time. After the process, the sample was filtered using a Buchner funnel and a hard filter. The obtained filtrates were tested for the content of Pb ions with the use of an atomic absorption spectrometer in an acetylene-air flame (F-AAS). All experiments were repeated 3 times and the results were averaged.

### 2.5. Statistical Analysis of the Fitted Models

The estimation of parameters in all models (equilibrium and kinetic) was made using the nonlinear regression method. The determination coefficient (R^2^) and the average relative error (ARE) were used to compare the quality of the results calculated from Equations (2) and (3) in Table 1 were used.

### 2.6. Equilibrium Studies

After equilibrium was reached at a constant temperature, the relationship between the amount of sorbate adsorbed by the sorbent and the concentration of sorbate remaining in the solution after reaching equilibrium was described by adsorption isotherms. The parameters of the models obtained in this way provide a lot of useful information regarding the properties of the sorbent surface, sorption mechanisms and mutual interactions between the sorbent and the sorbate [30]. Many models describe the adsorption equilibrium; however, the main ones used are the Langmuir and Freundlich models, while the literature on the subject also includes the Temkin and Dubinin–Radushkevich (D–R) models. Table 2 presents the equations of the used equilibrium models.

### 2.7. Kinetic Studies

Conducting kinetic tests is very important in the processes of water and wastewater treatment due to the information obtained in this way, including the mechanism of the sorption process or the optimal time of the process. The most popular kinetic models are the pseudo-first-order model, also called the Lagergren model, and the pseudo-second-order model, invented by Ho [35]. Additionally, you can also find the use of the Elovich and Weber–Morris models. These equations take into account the sorption capacity of the adsorbents and are presented in Table 3.

Based on the obtained results, the order of the reaction, the reaction rate constant and the optimal process time can be determined. The most effective removal of metals from a solution occurs when the adsorption process equilibrium is established.

## 3. Results and Discussion

### 3.1. Characteristic of RA and RA-SC

**Figure 1:**
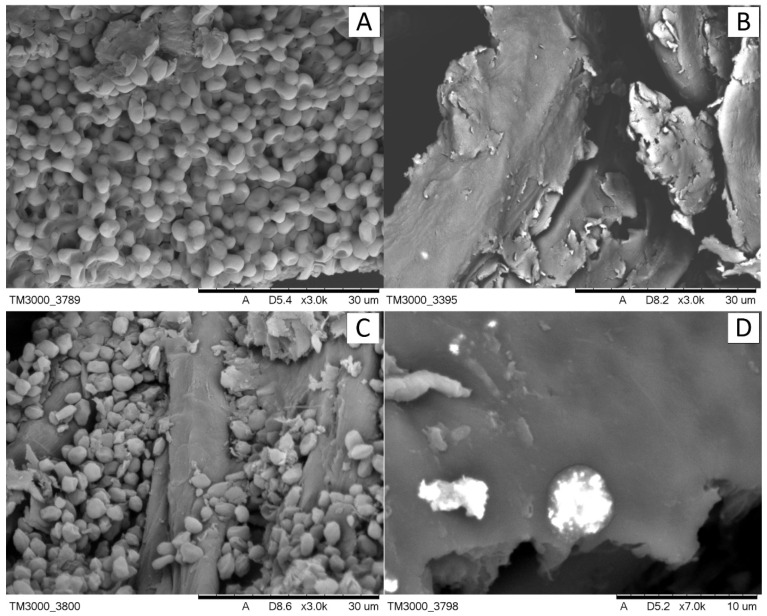
SEM microphotography: (**A**) SC, (**B**) RA, (**C**) RA-SC, (**D**) RA-SC-Pb.

In this study, we successfully obtained the RA-SC composite using *raphia* fibers as a carrier and immobilized the yeast *Saccharomyces cerevisiae* on its surface as a new biosorbent for removing lead ions from water and wastewater. Comparison of the surface morphology of SC, RA and RA-SC was performed using SEM (Figure 1). SC surface analysis showed that SC cells are characterized by a spheroid or slightly elongated spheroidal shape with regular and smooth surfaces. The regular surface area turned out to be very large and advantageous for the adsorption process (Figure 1A). SEM micrographs showed that the RA surface is heterogeneous and porous (Figure 1B). Additionally, BET analysis showed that the specific surface area of RA had a specific surface area of 1.079 m^2^/g, moreover, RA surface is characterized by a mesoporous structure. In Figure 1C, we can see SC cells on the surface of RA. The observed changes in morphology suggested that RA as a carrier is good support for SC cells. This confirms that the SC and RA cells were permanently attached after the immobilization process. In addition, an increase in the pore structure is observed, thanks to which ion transfer is possible, which increases the ability to remove impurities. The cell wall surface of one SC cell was not obscured by adjacent cells and thus was open to sufficient contact with lead ions, which increased the adsorption process. Figure 1D shows the changes in SC after the Pb(II) sorption process (RA-SC-Pb). In the photo, we can see that the shape of the cells has not changed much, and the presence of the removed lead ions is visible in the form of speckled particles distributed on the surface of the cells. Based on this, it can be assumed that the cell wall was the main part of the SC for Pb(II) adsorption. Similar observations were made by Zhang et al. in their research on the removal of heavy metal ions on yeast cells [40]. The EDX analysis of SC cells before sorption showed the presence of such elements as K, N, O, C, P and S, and additionally Pb after the process. Moreover, the presence of Pb on the RA surface was observed, and therefore these morphological changes can be attributed to the interaction of Pb(II) with RA-SC surface functional groups, which is consistent with the results of the FT-IR spectra analysis.

**Figure 2:**
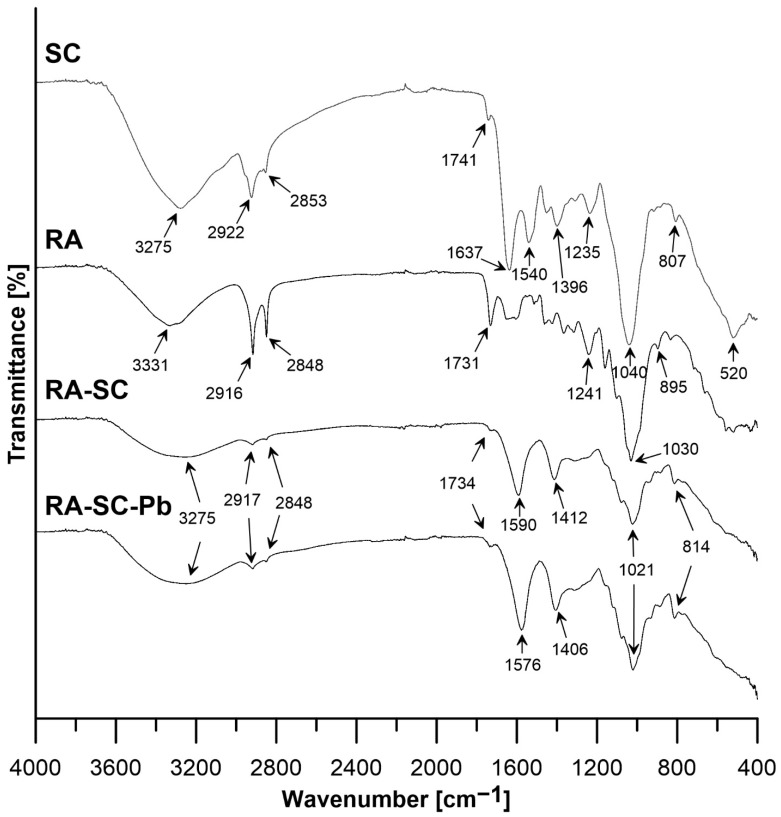
Results of FT–IR analysis SC, RA, RA-SC and RA-SC-Pb.

The performed analysis of FTIR SC, RA, RA-SC and RA-SC-Pb is shown in Figure 2. The analysis showed a difference in the chemical structure, the nature of bonds and interactions between the materials. Differences in frequency and intensity of the bands are observed, especially in the case of SC and other materials. The most intense broad band in the range of 2990–3600 cm^−1^ corresponds to the stretching vibrations of the hydroxyl groups -OH [41], in the range 2916–2922 cm^−1^ there are ν_asym_ CH_2_ (a large change in intensity and a shift of the peak in RA-SC can be observed before and after sorption in relation to SC and RA) and in the range of 2848 and 2853 cm^−1^ ν_sym_ CH_2_ (there is a change in the intensity of the peak in RA-SC before and after sorption against SC and RA) [42,43]; bands related to C–O bonding, C–N axial deformation, and primary amine bending NH_2_ were observed at 1637 cm^−1^ [44], stretching vibrations in lipid esters (-C=O) exist at the wavenumber 1734 cm^−1^ (shifted from 1741 cm^−1^ SC and 1731 cm^−1^ RA), the strong peak at 1040–1021 cm^−1^ corresponds to C–O stretching caused by glycosidic bonding, OH groups in sugars, or C–O–C stretching vibrations in lignin or hemicellulose, the δ C–H bonding of the aromatic ring, S=O stretching, 1060–1020 cm^−1^ [45,46,47]. Moreover, the relative intensity of the 1021 cm^−1^ band increases after the RA-SC-Pb adsorption process compared to RA-SC. Meanwhile, the absorption peak of -NH_2_ stretching shifted from 1396 to 1412 cm^−1^, and after sorption to 1406 cm^−1^, indicating that the amine group was involved in coordination with Pb(II), leading to an increase in the -NH_2_ bond length in the SC. The 1235 cm^−1^ peak is responsible for C=O vibrations [48], the anti-symmetric phosphate stretching band around 1241 cm^−1^ should therefore not be considered for estimation of lipid content [49]; the N–H and C–N vibrations of the peptide bond in different protein conformations 1590–1540 cm^−1^ (there is a shift of the peak maximum Δ$\bar{\upsilon }$ = 14 cm^−1^ after the Pb(II) sorption, indicating that nitrogen atoms are the adsorption sites for Pb(II) adsorption on RA-SC) [42,50]; 895–807 cm^−1^ represents the presence of sugars, mainly monosaccharides [51]; in the 600–400 cm^−1^ band, vibrations occur due to CNC deformation [52].

Based on the results of the FTIR analysis, it can be concluded that the observed spectral changes after Pb(II) sorption on RA-SC most likely result from possible changes in the chemical structure of the composite. Thus, the described spectral differences suggest the participation of the above-mentioned functional groups in the binding of Pb(II) to the RA-SC surface.

### 3.2. Effect of Time and Initial Concentration on Metal Ion Sorption

**Figure 3:**
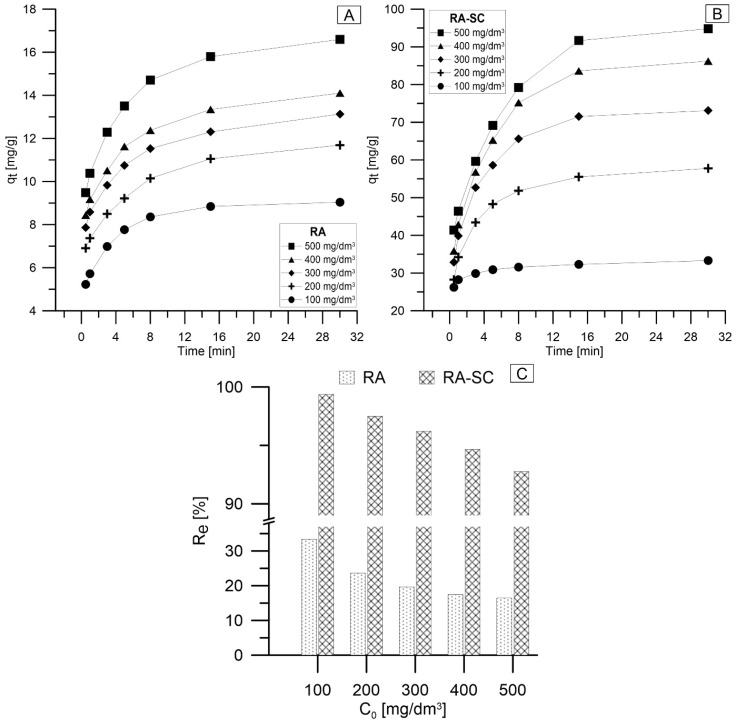
The sorption capacity in time depending on the initial concentration: (**A**) RA, (**B**) RA-SC, (**C**) the degree of metal removal from the RA and RA-SC model solutions (T = 25 °C, pH = 5).

The first stage of the sorption process is the determination of the time after which the equilibrium between the sorbent and the sorbate is established. Based on the experimental data, the equilibrium time was set at 30 min. Figure 3 shows the effect of the processing time on the sorption capacity of RA (Figure 3A) and RA-SC (Figure 3B). It was observed that the lead ion sorption process on RA and RA-SC consisted mainly of two stages. In the initial stage of the process, a rapid increase in sorption capacity was observed for both materials. It could be observed that the uptake of Pb(II) ions increased significantly early in the process, which was probably due to electrostatic attraction and a large amount of free active sites. The initial increase in q_t_ was related to the number of available active sites capable of binding lead ions. In the initial phase, the sorbent has a large number of active centers, and the ingredients are strongly adsorbed to the active centers [53]. The highest temporary increase in sorption capacity was observed within 3 min. For RA, the difference in q_t_ between 3 and 1 min varied from 1.1 mg/g (C_0_ = 100 mg/dm^3^) to 1.91 mg/g (C_0_ = 500 mg/dm^3^), and for RA-SC in in the range from 1.7 mg/g (C_0_ = 100 mg/dm^3^) to 13.9 mg/g (C_0_ = 500 mg/dm^3^). The second slower step occurred before the lead ion sorption reached equilibrium, which was a gradual adsorption step. With the time the process is carried out, the difference between q_t_ and the following times decreases until an equilibrium is established. Additionally, it can be observed that an increase in the initial concentration of lead ions causes an increase in the value of the sorption capacity. This is due to the higher number of lead ions, which also translates into an increase in the number of collisions between them and the active centers on RA and RA-SC [54]. In addition, it can be observed that the curves of the lead ion removal process are more vertical in the case of RA-SC, which is related to its better ability to remove the studied ions. The maximum sorption capacity obtained for the removal of Pb(II) on RA was 16.6 mg/g and for RA-SC it was 94.8 mg/g as a function of time. Moreover, the present study showed that the maximum adsorption capacity of RA-SC was significantly higher (almost 6-fold higher) than that of RA. The difference in sorption capacity between RA and RA-SC is related to the activity of *Saccharomyces cerevisiae* yeast and the method of immobilization of microorganisms (the material after immobilization additionally contained calcium alginate). The difference in the initial and final sorption capacities in the materials is a direct result of their composition. The RA material is *raphia* fibers that have not undergone any modifications, so in the case of RA responsible for the lead ion sorption process are cellulose, hemicellulose, lignin, extracts, and many other compounds, such as lipids, starch, hydrocarbons, simple proteins and ash [55]. Organic compounds (lignin, cellulose and hemicellulose) with polyphenolic groups could bind heavy metal ions through different mechanisms [56]. The ability to remove heavy metal ions by such biosorbents (without modification) is much lower than after modification. Wu et al. in their research found that the sorption capacity in relation to Pb(II) of unmodified biosorbents was equal to: cotton—10.78 mg/g, wood sawdust—17.03 mg/g, buckwheat hull—34.06 mg/g [57]. RA-SC achieved excellent contact with the solution and lead ions by increasing the pore structure and the fact that the SC cell walls were not obstructed by adjacent cells. RA-SC achieved excellent contact with the solution and lead ions by increasing the pore structure and the fact that the SC cell walls were not obstructed by adjacent cells. According to Zhang et al., immobilization of yeast cells increased the sorption capacity by almost 60% [40]. Other researchers in a study of lead and cadmium ion removal by EDTAD-functionalized *Saccharomyces cerevisiae* sorbent came to similar results. They observed that the removal of lead ions was very fast, in the first 5 min of the process, almost 90% of adsorption took place, and after 30 min it reached the equilibrium state (q_e_ = 83.99 mg/g) [6]. This type of adsorption is typical for surface adsorption behavior, the adsorption capacity of which depends on the number of available adsorption sites on the adsorbent surface [58]. The high adsorption capacity and short adsorption equilibrium time showed that the RA-SC surface has a high density of functional groups. In addition, the reason for determining the adsorption equilibrium time within 30 min may be the gradual penetration by lead ions of the outer structural layer of the yeast cell wall (due to the functional groups present therein), which was destroyed by the vacuum freeze-drying step in the RA-SC preparation processes. Figure 3C shows the rates of lead ion removal by RA and RA-SC. The value of R_e_ decreases with the increase of the initial concentration of Pb ions, which is directly related to the affinity of the tested materials for Pb(II). For RA, a change in R_e_ was observed from 33.5% to 16.5% (a difference of 17 pp), with the difference between the individual concentrations of C_0_ decreasing by 6 pp to 1 pp, and for RA-SC, the change in R_e_ was about 5 pp, from ~98% to ~93%, with the R_e_ difference between the individual concentrations of C_0_ being low, in the range of 1–1.9%. This was confirmed by the fact that RA-SC has higher Pb(II) removal abilities than RA.

### 3.3. Equilibrium Studies

**Figure 4:**
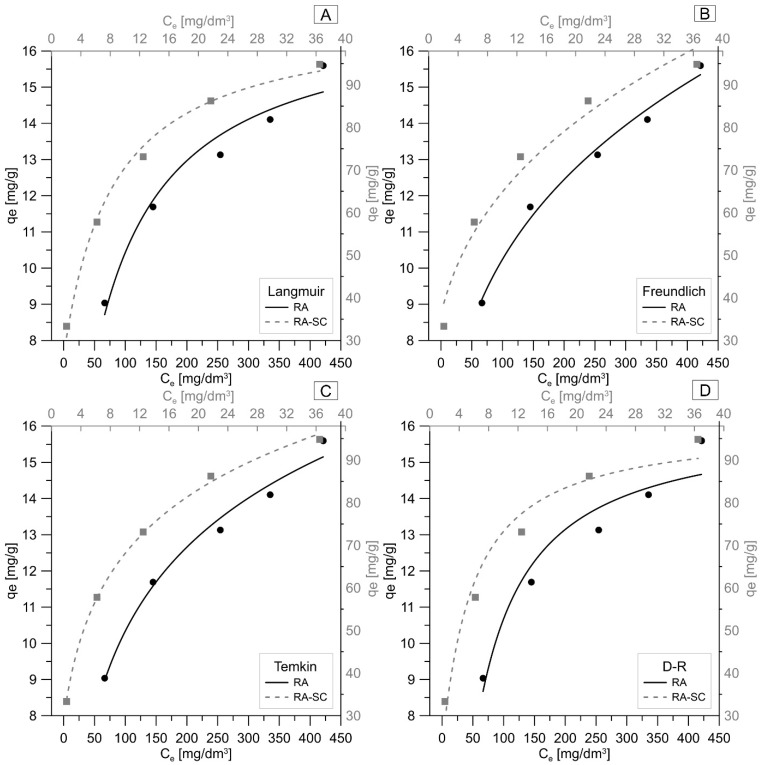
Graphic representation of sorption isotherms RA and RA-SC: (**A**) Langmuir, (**B**) Freundlich, (**C**) Temkin, (**D**) D-R (T = 25 °C, pH = 5).

To learn the nature of the sorption process, equilibrium and kinetic studies were used. Four isotherm models were investigated in the study: Langmuir, Freundlich, Tempkin and Dubinin–Radushkevich. Figure 4 and Table 4 show the obtained results of the equilibrium tests. To select the isotherm model that best describes the process of removing Pb ions on RA and RA-SC, the average relative error ARE and the correlation coefficient R^2^ were used. It can be observed that the type of sorbent affects the course of sorption. The Temkin model is the model that best describes the process of Pb(II) removal by RA and RA-SC. For RA, ARE was 1.77% and R^2^ = 0.9884, while for RA-SC, ARE was 0.95% and R^2^ = 0.9984. Additionally, high matches were found for the Langmuir model for RA-SC (ARE = 2.82%, R^2^ = 0.9934) and the Freundlich model for RA (ARE = 1.80%, R^2^ = 0.9839). The Temkin isotherm contains a factor that clearly takes into account the adsorbent–adsorbate interactions. It is assumed that due to these interactions and the omission of very low and very high concentration values, the heat of adsorption of all molecules in the layer would decrease linearly with the coverage [59]. The values obtained from the Temkin isotherm for RA were: K_T_ = 0.22 dm^3^g^−1^ and B = 3.3 kJmol^−1^, which indicates that adsorption of Pb(II) on RA takes place by physisorption. The binding energy value was <8 kJmol^−1^, thus physical adsorption is the mechanism involved. In the physiadsorption process, adsorbates adhere to the adsorbent through weak van der Waals interactions, and thus this process is associated with relatively low adsorption energies [60]. However, in the case of RA-SC, the obtained values were K_T_ = 2.3 dm^3^g^−1^ and B = 21.7 kJmol^−1^, which proves in this case that we are dealing with chemisorption. Based on the Freundlich isotherm for RA, the calculated parameter 1/n was 0.28, which allows us to conclude that RA is a suitable sorbent for Pb(II) removal (value 1/n < 1) [61]. The Freundlich isotherm predicts infinite surface coverage and therefore does not predict the saturation of the material by the sorbed compound (multi-layer adsorption model). The calculated parameter n = 3.57, which is in the range of 1 < n< 10, indicates favourable sorption conditions [62]. Langmuir adsorption isotherm is based on the assumption that all sorption sites are equivalent and there is no dependence of the sorption sites on the occupied neighboring sites [63]. The theoretical saturation capacity of the monolayer is 109.1 mg/g (RA-SC). The fact that the experimental data fit well with the Langmuir isotherm may be due to the homogeneous distribution of active centers on the RA-SC surface, since the Langmuir equation assumes that the surface is homogeneous [64]. Similar results from their research on Pb(II) removal on activated carbon obtained from apricot kernels were obtained by Mouni et al. [65]. In the case of these studies, the Langmuir isotherm was the best fit of all the isotherms tested, and the calculated separation coefficient also indicated favorable sorption conditions.

### 3.4. Kinetic Studies

**Figure 5:**
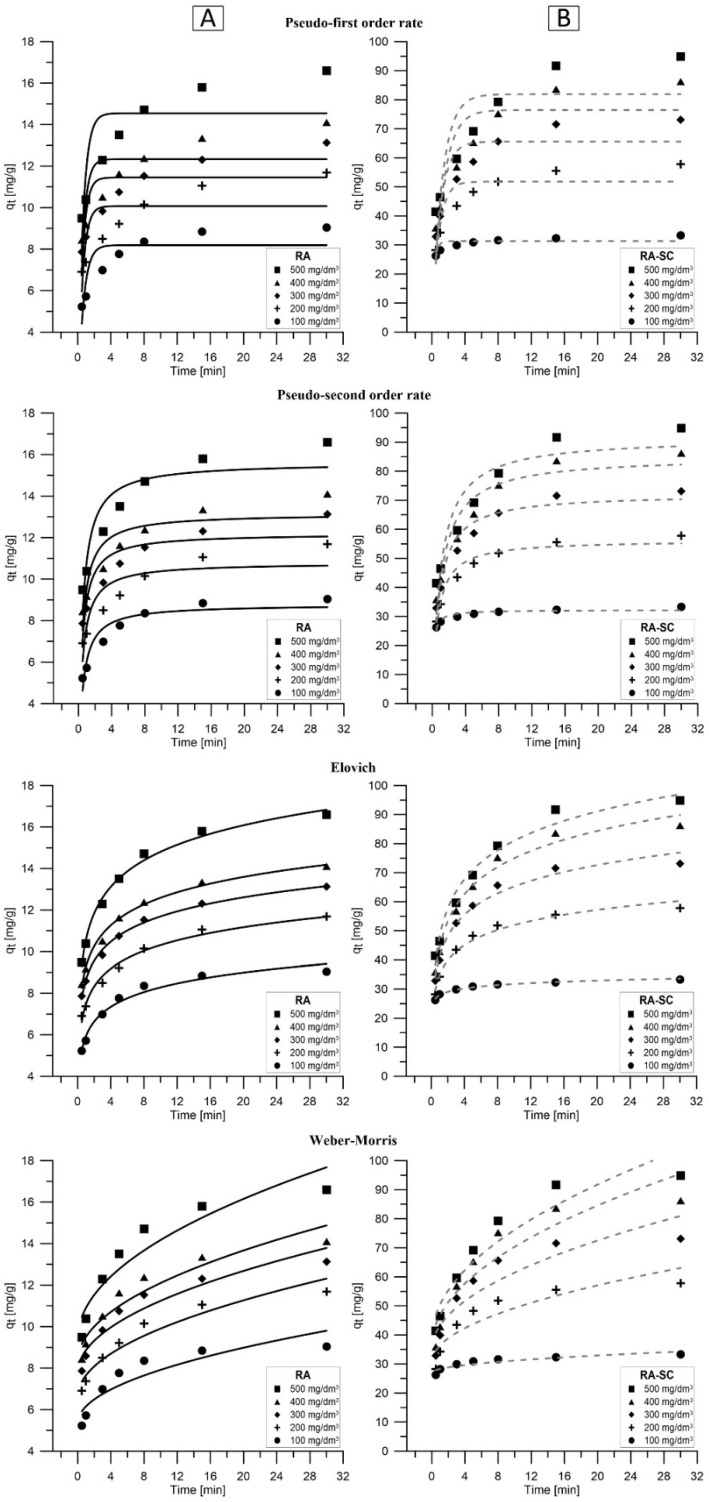
Graphic representation of pseudo-first-order, pseudo-second-order, Elovich and Weber–Morris models for the process of ions adsorption: (**A**) RA, (**B**) RA-SC (T = 25°C, pH = 5).

Many physicochemical factors can limit the speed of the sorption process. One of the main factors is the interaction between the sorbate and the sorbent surface. Both penetration into the pores and the deposition of sorbate on the sorbent surface affect the sorption rate. The obtained kinetic results presented in Figure 5 and Table 5 show that in the case of RA, the ratio of q_t_ in 30 min to q_t_ does not change during 0.5 min during the change of the initial concentration of Pb(II), which is 1.73 for C_0_ = 100 mg/dm^3^ and 1.75 for C_0_ = 500 mg/dm^3^. However, in the case of RA-SC, this difference is significant, and amounts to 1.27 for C_0_ = 100 mg/dm^3^ and 2.29 for C_0_ = 500 mg/dm^3^. It is related to the number of moles of Pb(II) in the solution. Initially, there is a large number of sorbate moles in the solution, which means that the filling of free active centers is associated with a lower driving force resulting from a smaller number of active collisions between Pb(II) and RA and RA-SC. On the other hand, with an increase in the initial concentration, a greater number of active collisions is observed, which translates into a greater initial q_t_ [54]. Additionally, it was observed that depending on the type of sorbent, the ratio of sorption capacity changes at the initial concentration of Pb(II). For RA this ratio is 1.84 and for RA-SC it is 2.85, which proves that RA-SC has higher lead ion removal capacity and therefore an increase in C_0_ concentration translates into an increase in q_e_.

Kinetic studies were carried out for four kinetic models: pseudo-first-order, pseudo-second-order, Elovich and Weber–Morris. The performed calculations allowed for the conclusion that the model that best describes the process of removing lead ions by RA and RA-SC is the Elovich model. For RA, the ARE ranged from 1.02% to 2.35% and for RA-SC from 0.94 to 4.18%. In contrast, R^2^ was in the range of 0.97–0.99% for RA and ~ 0.98% for RA-SC. The Elovich model assumes that there are no interactions between the adsorbed species and that the actual solid sorbent surface is energetically heterogeneous [66]. The α and β coefficients appearing in the Elovich equation are related to the initial adsorption rate and the desorption coefficient. The observed value of the α coefficient higher than β indicates that the dominant process is adsorption then desorption [67]. The 1/β value reflects the number of sites available for adsorption [68]. Additionally, based on the Elovich model, it was found that with the increase of C_0_, the share of diffusion as the driving force of the Pb(II) removal process increases. It follows that lower concentrations of C_0_ favor the processes of chemical adsorption and intramolecular diffusion, and at higher concentrations, the activation energy is higher, which means that they share in chemical sorption is higher, which increases the sorption capacity [69]. The compliance of the Elovich equation with the experimental data obtained for RA (for which the information obtained in the equilibrium studies that physisorption is responsible for the Pb(II) removal process) is related to the heterogeneity of the RA structure. The heterogeneity of RA is associated with a change in the chemisorption energetics with active centers. In heterogeneous material, active centers show different activation energies for chemisorption, which is directly related to the presence of cellulose, lignin, hydroxyl groups, tannins and phenolic compounds [70,71]. The Elovich model is characterized by a good fit to the data obtained for sorption on highly inhomogeneous surfaces and shows that apart from surface adsorption, chemisorption is also the dominant phenomenon. On such a heterogeneous material, apart from surface adsorption, additionally, chemisorption, precipitation, intramolecular diffusion and ion exchange are observed, which occur simultaneously. The Elovich equation does not predict any particular mechanism, but is useful in describing adsorption on highly heterogeneous adsorbents. Similar conclusions were reached by Riahi et al. in their studies of phosphate removal on date palm fibers [72]. Additionally, it was found that the constant α related to the rate of chemisorption increases with the increase of the initial concentration of Pb(II), which suggests that more than one mechanism regulates the removal of the studied ions [73].

## 4. Conclusions

The conducted research allows us to state that it was possible to obtain a composite biosorbent for removing lead ions from water and sewage. The obtained composite consisting of *raphia* fibers as a carrier and immobilized *Saccharomyces cerevisiae* yeast cells was characterized by high Pb(II) removal capacity. The characterization of the biosorbent after the sorption process confirmed the presence of the tested ions on its surface. The obtained RA-SC composite had a greater affinity for Pb(II) by more than 5.7× compared to RA (94.8 mg/g—RA-SC 16.6 mg/g—RA). The equilibrium model that best describes the lead ion removal process by RA-SC and RA is the Temkin isotherm model (RA: ARE = 1.77%, R^2^ = 0.9884, RA-SC: ARE = 0.95%, R^2^ = 0.9984). The performed kinetic studies made it possible to state that the Elovich model best describes the process of Pb(II) removal by RA and RA-SC (ARE < 4.18% and R^2^ > 0.97). Moreover, compared to chemical procedures, the RA-SC synthesis procedure is simple and inexpensive. Thus, RA-SC may be a promising candidate for the removal of lead or other heavy metal ions from water and wastewater.

## Figures and Tables

**Table 1:** The equation used in the presented study.

Isotherm Models	Equation	Reference	No. Equation
Sorption capacity	$q_{e}=\frac{\left(C_{0}-C_{e}\right)\cdot V}{m}$	[24]	(1)
The determination coefficient	$R^{2}=1-\frac{\sum_{1}^{n}{\left(q_{\exp}-q_{\mathrm{pred}}\right)}^{2}}{\sum_{1}^{n}{\left(q_{\exp}-\bar{q_{\exp}}\right)}^{2}}$	[25]	(2)
The average relative error	$\mathrm{ARE}=\frac{100}{n}\overset{n}{\underset{1}{\sum }}\frac{\left|q_{\exp}-q_{\mathrm{pred}}\right|}{q_{\exp}}$	[26]	(3)

**Table 2:** Nonlinear equations of isotherm models.

Isotherm Models	Equation	Reference	No. Equation
Langmuir	$q_{e}=\frac{q_{m}K_{L}C_{e}}{1+K_{L}C_{e}}$	[31]	(4)
Freundlich	qe=KFCe1n	[32]	(5)
Temkin	$q_{e}={\mathrm{BlnK}}_{T}C_{e}$	[33]	(6)
Dubinin–Radushkevich	$q_{e}=q_{d}\exp\left(-K_{ad}\varepsilon^{2}\right)$	[34]	(7)
	$\varepsilon =\mathrm{RTln}\left(1+\frac{1}{C_{e}}\right)$		(8)

**Table 3:** Nonlinear kinetic equations.

Kinetic Models	Equation	Reference	No. Equation
Pseudo-first-order	$q_{t}=q_{1}\left(1-\exp\left(-k_{1}t\right)\right)$	[36]	(9)
Pseudo-second-order	$q_{t}=\frac{t}{(1/k_{2}q_{2)}^{2}+\left(t/q_{2}\right)}$	[37]	(10)
Elovich	$q_{t}=\frac{1}{\beta }\ln\left(1+\alpha \beta t\right)$	[38]	(11)
Weber–Morris	$q_{t}=K_{id}\sqrt{t}+I$	[39]	(12)

**Table 4:** Parameters of sorption isotherms models.

Isotherm Model	Parameters				
Langmuir		ARE [%]	R^2^	q_m_ [mg/g]	K_L_ [dm^3^/mg]
	RA	3.18	0.9583	17.139	0.0155505
	RA-SC	2.82	0.9934	106.089	0.1985247
Freundlich		ARE [%]	R^2^	K_F_ (mg^1−(1/n)^(dm^3^)^1/n^g^−1^)	1/n
	RA	1.80	0.9839	2.81066	0.28086
	RA-SC	6.49	0.9705	30.61679	0.32560
Temkin		ARE [%]	R^2^	K_T_ [dm^3^/g]	B
	RA	1.77	0.9884	0.2194991	3.347
	RA-SC	0.95	0.9984	2.2944000	21.711
D-R		ARE [%]	R^2^	K_ad_ [mol^2^/kJ^2^]	q_d_ [mg/g]
	RA	3.99	0.9353	0.0171906	16.199
	RA-SC	6.36	0.9683	0.0012690	98.249

**Table 5:** Parameters of sorption kinetic models for the sorption process Pb ions.

Kinetic Model		Lead Ion Concentration C_0_ [mg/dm^3^]				
		100	200	300	400	500
Pseudo-first-order rate						
RA	q_1_ [mg/g]	8.190	10.073	11.452	12.334	14.547
	k_1_ [min^−1^]	1.5451	1.7929	1.8558	1.8258	1.6270
	R^2^	0.7189	0.5612	0.6158	0.6137	0.6502
	ARE [%]	9.80	11.19	9.63	9.82	10.53
RA-SC	q_1_ [mg/g]	31.328	51.796	65.565	76.454	81.915
	k_1_ [min^−1^]	3.3515	1.2194	0.9935	0.7753	0.8181
	R^2^	0.6748	0.8125	0.8051	0.7740	0.6878
	ARE [%]	3.70	9.02	10.50	13.25	14.94
Pseudo-second-order rate						
RA	q_2_ [mg/g]	8.777	10.789	12.197	13.153	15.618
	k_2_ [min^−1^]	0.2534	0.2354	0.2258	0.2041	0.1465
	R^2^	0.9088	0.7963	0.8387	0.8382	0.8623
	ARE [%]	5.56	7.65	6.21	6.43	6.57
RA-SC	q_2_ [mg/g]	32.222	56.295	72.189	85.027	91.513
	k_2_ [min^−1^]	0.2445	0.0290	0.0176	0.0117	0.0111
	R^2^	0.9074	0.9554	0.9482	0.9270	0.8754
	ARE [%]	1.94	4.54	5.57	7.52	9.73
Elovich						
RA	β [g/mg]	0.9763	0.8080	0.7539	0.6908	0.5424
	α [mg/g·min]	332.8	514.7	892.4	866.1	566.0
	R^2^	0.9753	0.9830	0.9948	0.9925	0.9915
	ARE [%]	2.35	2.21	1.02	1.32	1.64
RA-SC	β [g/mg]	0.5966	0.1325	0.0940	0.0735	0.0685
	α [mg/g·min]	28,433,115	737.9	487.4	334.8	373.3
	R^2^	0.9825	0.9818	0.9801	0.9825	0.9772
	ARE [%]	0.94	2.62	2.74	3.01	4.18
Weber-Morris						
RA	I	5.3411	6.6159	7.7652	8.3106	9.3924
	K_id_	0.8144	1.0401	1.1002	1.1985	1.5125
	R^2^	0.8300	0.9353	0.9206	0.9153	0.8989
	ARE [%]	7.56	4.15	4.47	4.67	5.71
RA-SC	I	26.9339	30.4228	34.8629	35.9809	38.5482
	K_id_	1.3367	5.9555	8.4153	10.8642	11.9007
	R^2^	0.8393	0.8266	0.8358	0.8714	0.9086
	ARE [%]	2.57	9.45	10.60	10.14	7.79

## Data Availability

Not applicable.
